# Successful Dichoptic Therapy for Amblyopia in a Child Unable to Tolerate Occlusion Therapy due to Autism Spectrum Disorder

**DOI:** 10.1155/crop/9634126

**Published:** 2026-03-11

**Authors:** Shuji Nakatsuka, Yo Iwata, Hirotaka Ito, Yuta Nakano, Kumiko Mokuno, Tomoya Handa

**Affiliations:** ^1^ Department of Ophthalmology, Kariya Toyota General Hospital, Kariya, Japan, toyota-kai.or.jp; ^2^ Department of Vision Science, Kitasato University Graduate School of Medical Sciences, Sagamihara, Japan, kitasato-u.ac.jp; ^3^ Department of Rehabilitation, Orthoptics and Visual Science Course, School of Allied Health Sciences, Kitasato University, Sagamihara, Japan, kitasato-u.ac.jp

**Keywords:** amblyopia, autism spectrum disorder, dichoptic therapy, Occlu-pad

## Abstract

Children with autism spectrum disorder (ASD) often face challenges with occlusion therapy using eye patches owing to the sensory sensitivity, rigid behaviors, and communication difficulties associated with ASD. In this study, we report the case of a child with ASD who had significant difficulty with eye patch therapy for anisometropic amblyopia and was treated with visual acuity training using Occlu‐pad, a dichoptic therapy device. This treatment led to marked improvement in visual acuity and high compliance. After 3 months of home‐based therapy for 30 min per day, the best corrected visual acuity in the right eye improved from logarithm of minimum angle of resolution of 0.5 to 0.0, with an average compliance rate of 92%. This case highlights the importance of flexible treatment approaches tailored to individual patient characteristics in managing amblyopia in children with ASD and also demonstrates the potential utility of dichoptic therapy using Occlu‐pad.

## 1. Introduction

Autism spectrum disorder (ASD) is a neurodevelopmental disorder characterized by difficulties in social communication and restricted repetitive behavioral patterns. It has a reported prevalence of 1%–2% in the general population [1, 2]; however, recently, the number of individuals diagnosed with ASD has increased [3]. The ocular manifestations associated with ASD include amblyopia, anisometropia, astigmatism, hyperopia, exotropia, and esotropia [4, 5]. Accordingly, individuals with ASD are often reported to have multifaceted visual dysfunction not limited to reduced visual acuity, including deficits in binocular vision, stereopsis, eye movements, and visual information processing; therefore, comprehensive evaluation of visual function is recommended [6, 7]. The treatment of amblyopia during childhood typically involves corrective glasses and occlusion therapy; however, due to the strong preferences and sensory sensitivities commonly observed in ASD, it is often challenging for healthcare providers to gain patients′ cooperation during examinations and treatment. Despite the elevated risk of visual dysfunction in ASD, the rate of vision screening in primary care has been reported to be low [8].

Full‐time wearing of spectacles with complete refractive correction is the first‐line treatment for amblyopia [9]. Nevertheless, in moderate amblyopia, approximately only 30% of patients reportedly achieve a best corrected visual acuity (BCVA) (expressed as logarithm of the minimum angle of resolution [logMAR] value) of 0.1 or better with refractive correction alone [10]. Therefore, occlusion therapy, which involves covering the nonamblyopic eye with a patch to force the patient to use the amblyopic eye, is generally required in addition [11]. However, maintaining compliance is often difficult because children may remove the patch during treatment. Reportedly, compliance with occlusion therapy is typically less than 50% during treatment, and it declines over time [12].

In response to these challenges, dichoptic therapy has recently been developed and implemented as an alternative approach to occlusion therapy for amblyopia [13, 14]. One of the devices that enable dichoptic therapy is Occlu‐pad [15], a tablet‐based device that employs white‐screen technology to facilitate dichoptic training. The device allows selective visual stimulation of the amblyopic eye under binocular viewing conditions, and it is used by removing the polarizing film layer from the liquid‐crystal display panel and attaching it to specialized glasses (Figure 1).

FIGURE 1External view and viewing mechanism of Occlu‐pad. (a): External appearance of the Occlu‐pad device. (b): View of Occlu‐pad when a polarized film is applied over the right eye, illustrating how the image becomes visible only through the polarized lens.

**Figure:**
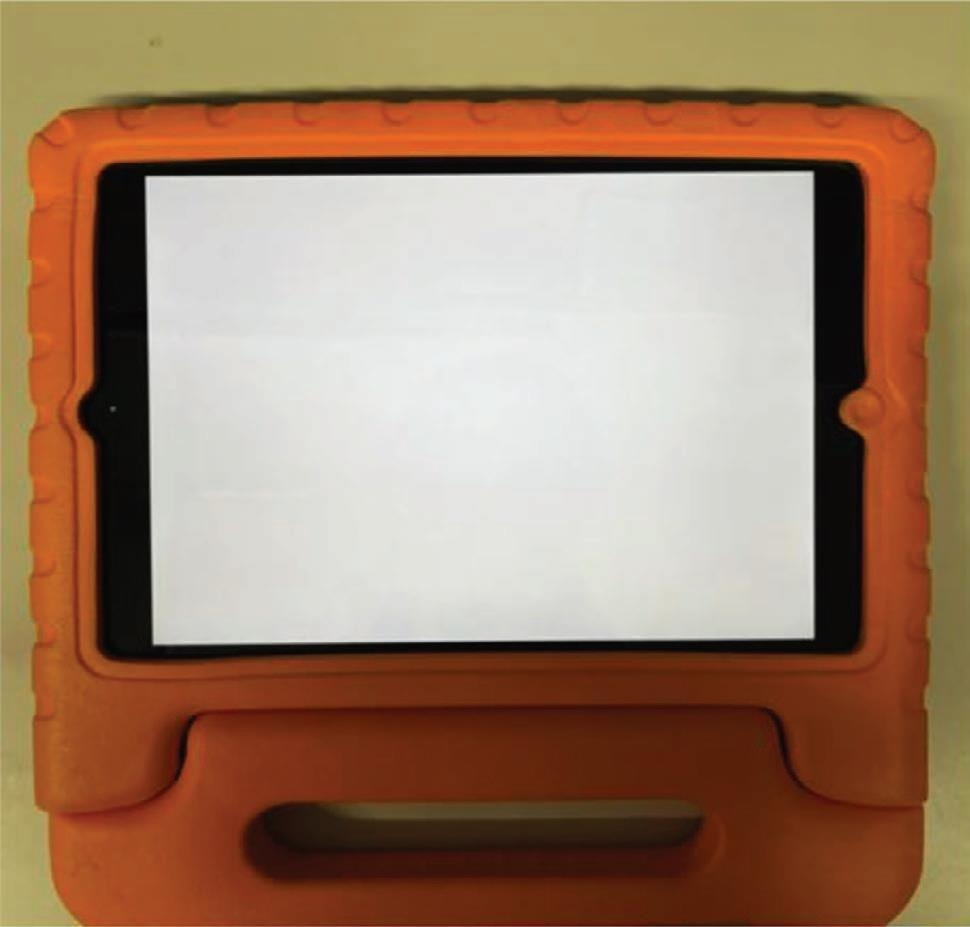
(a)

**Figure:**
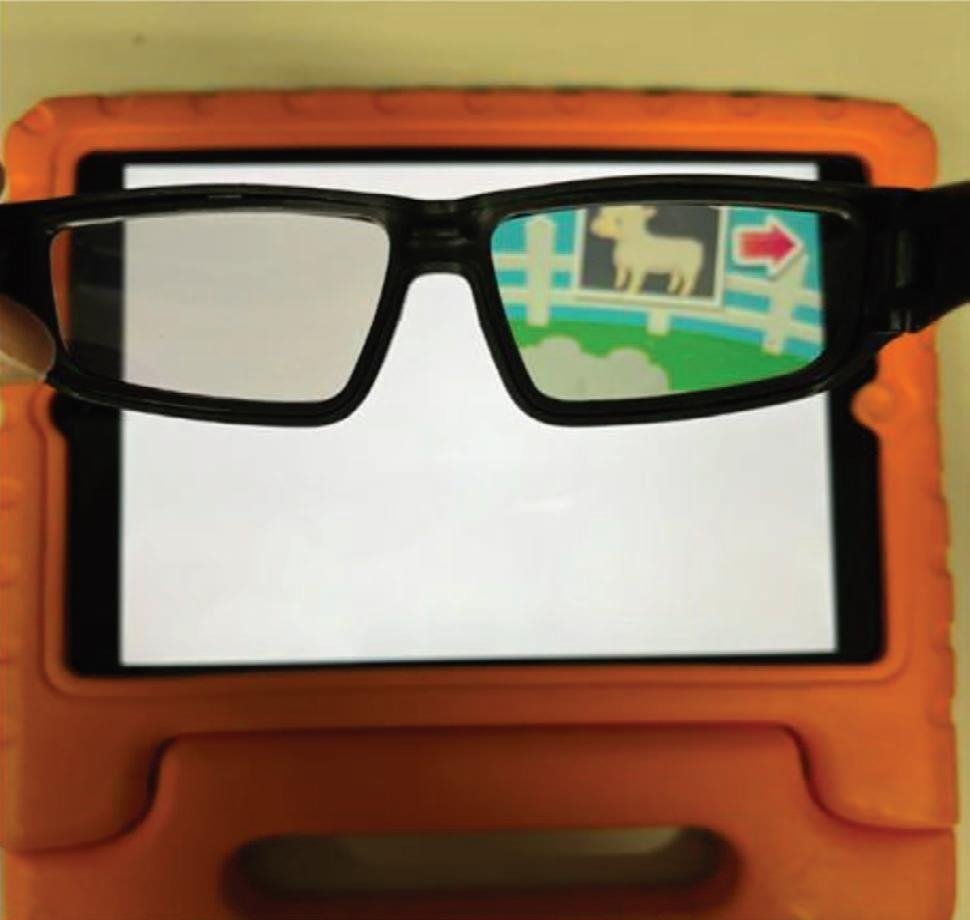
(b)

The use of games has been reported to be effective in promoting social skills, emotional understanding, attention, and flexible thinking in individuals with ASD, which is characterized by difficulties in participating in communities and social activities [16]. Since Occlu‐pad is based on an iPad platform, treatment can be customized to the patient′s preferences using various game applications and video content. Furthermore, as polarized glasses are required for the screen to be visible, the device promotes active engagement in amblyopia therapy [15]. Although low compliance is a common issue with patching therapy, patients who use Occlu‐pad have been shown to maintain high levels of compliance during training sessions [17].

Here, we report the case of a child with ASD and anisometropic amblyopia in whom traditional occlusion therapy using an eye patch was not feasible. The patient was successfully treated with dichoptic therapy using Occlu‐pad.

## 2. Case Report

The patient is a 7‐year, 9‐month‐old girl with ASD, diagnosed by a pediatrician in early childhood. In April 2021, she was diagnosed with anisometropic amblyopia in the right eye. In May 2021, fully refractive spectacles were prescribed after cycloplegic refraction using cyclopentolate hydrochloride. The spectacle prescription was +5.50 diopters (D), cyl −1.50 D axis 5° in the right eye, and +1.50 D in the left eye. Her BCVA was 0.7 logMAR in the right eye and 0.0 logMAR in the left eye. Therefore, corrective glasses and occlusion therapy were recommended. However, the patient strongly refused to undergo occlusion therapy with an eye patch, rendering it unfeasible.

After 11 months of regular spectacle use, the BCVA in the right eye only improved slightly, reaching 0.5. Ocular alignment revealed mild exophoria at both distance and near fixation, with and without spectacles. No abnormality in ocular motility was observed.

In May 2022, follow‐up cycloplegic refraction was performed using atropine sulfate hydrate, and new spectacles were prescribed. The updated prescription was +6.00 D, cyl −2.00 D axis 175° in the right eye, and +2.00 D in the left eye. Occlusion therapy was attempted again but remained unfeasible because of persistent refusal by the patient.

Given these circumstances, dichoptic visual training using Occlu‐pad (with both eyes open) was proposed. After obtaining informed consent from both the patient and her guardian, treatment was initiated in May 2022.

The patient was instructed to use Occlu‐pad at home for 30 min daily. The treatment consisted of viewing selected games and videos available on App Store. During the treatment period, parents were asked to record the actual training time daily, and compliance was monitored.

**FIGURE 2:**
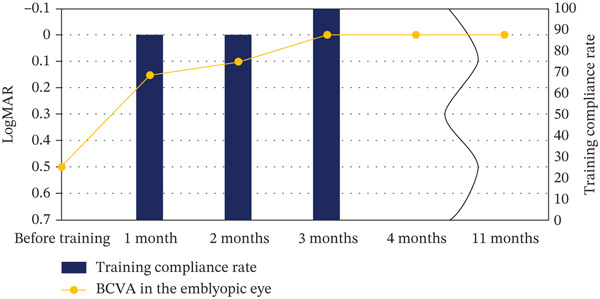
Changes in BCVA of the amblyopic right eye and Occlu‐pad compliance rate during the treatment period. Graph showing improvement in the BCVA of the right (amblyopic) eye over the course of treatment and the corresponding compliance rate with Occlu‐pad usage throughout the training period. Three months after initiating training with the Occlu‐pad, BCVA reached 0.0 logMAR and was maintained throughout the subsequent follow‐up. BCVA, best corrected visual acuity; logMAR, logarithm of the minimum angle of resolution.

After 1 month of Occlu‐pad use, the BCVA in the right eye improved to 0.15 logMAR in June 2022, to 0.1 logMAR in July 2022 (2 months later), and remained at 0.0 logMAR in August 2022 (3 months after treatment initiation) (Figure 2). By September 2022 (4 months after treatment was commenced), the BCVA in the right eye was maintained at 0.0 logMAR, and Occlu‐pad therapy was therefore concluded. Compliance rates were 88% after 1 month, 87% after 2 months, and 100% after 3 months, averaging 92% over the treatment period (Figure 2). No significant side effect or treatment rejection was observed. As the patient gradually became accustomed to the hospital environment and examination procedures, stereopsis testing became feasible at 8 months after treatment initiation. At that time, good stereoacuity of 20 arcsec was achieved, as assessed by the Randot Stereo Test, a forced‐choice task with three options (chance level 33%). Stereoacuity was maintained at the 11‐month follow‐up, with 20 arcsec confirmed using the same test. BCVA in the right eye remained at 0.0 logMAR.

## 3. Discussion

In this case, conventional occlusion therapy using an eye patch was not feasible for treating anisometropic amblyopia in a child with ASD due to several ASD‐related challenges, including sensory hypersensitivity, cognitive rigidity, resistance to change, communication difficulties, limited understanding of treatment goals, and behavioral issues associated with anxiety and stress [1–3]. Nevertheless, significant improvement in visual acuity was achieved through dichoptic treatment using Occlu‐pad.

Occlusion therapy, which involves patching the dominant eye to stimulate the amblyopic eye, is often limited by patient′s resistance as it requires the patient to perform visual tasks using the weaker eye, which can cause discomfort and noncompliance [12]. Additionally, skin irritation and discomfort caused by adhesive patches have been reported [18]. Individuals with ASD often exhibit sensory hypersensitivity and sensory‐processing difficulties that can lead to increased sensitivity to the tactile stimulation and pressure of eye patches, resulting in considerable stress. Furthermore, due to the strong resistance to change and rigid behavioral patterns commonly observed in people with ASD [1–3], the visual changes associated with patching can also induce considerable psychological distress. Moreover, children with ASD may struggle to comprehend the purpose of the treatment, making consistent use of the patch difficult; many remove it shortly after its application. Consequently, the likelihood of occlusion therapy being ineffective or entirely unfeasible is higher in this population than in their typically developing peers. Given these challenges, amblyopia treatment in children with ASD requires strategies that sustain interest and promote engagement. In previous reports, it has been reported that applying games is effective in assessing ASD development [19] and improving social, emotional, and communication skills [20].

The Occlu‐pad system is uniquely designed such that the screen is only visible when polarized glasses are worn [21]. In this case, the child was highly motivated to wear polarized glasses to watch preferred videos and play games, which encouraged voluntary and consistent participation in the amblyopia treatment. As a result, Occlu‐pad proved to be a highly suitable therapeutic option for this patient. These findings suggest that integrating game‐based elements into amblyopia therapy is a promising and engaging approach.

Furthermore, per recent randomized controlled trials, dichoptic treatment is comparable to, or even more effective than, traditional occlusion therapy [22, 23]. Occlu‐pad allows flexible and developmentally appropriate therapeutic engagement through a variety of interactive content, including video viewing, drawing, and gaming.

Stereopsis is an important binocular outcome in amblyopia and should be evaluated whenever feasible, both before and after treatment. In anisometropic amblyopia, stereoacuity often remains reduced compared with age‐matched norms even after successful treatment, and better stereoacuity outcomes are associated with smaller amounts of anisometropia [24]. In the present case, stereopsis could not be measured before amblyopia treatment owing to limited cooperation. The Randot circles task is a three‐alternative forced‐choice test (chance level 33%), and occasional correct responses may occur by chance. Given the magnitude of anisometropia in this case, the possibility that the observed correct responses reflected chance performance rather than true stereoacuity cannot be excluded. Despite these limitations, stereopsis testing is quick and practical in routine clinical settings and remains useful for screening and longitudinal monitoring of binocular function.

In this case, the improvement in visual acuity can be attributed to three key factors: (1) the motivational aspect of gameplay, (2) avoidance of previously unsuccessful experiences with occlusion therapy, and (3) the inherent effectiveness of dichoptic treatment. The child′s mother reported high compliance, strict adherence to the prescribed training schedule, and a positive attitude toward treatment. Based on these observations, dichoptic treatment using Occlu‐pad appears to be a highly useful and effective approach for treating amblyopia in children with ASD. Further research, including large observational studies and prospective trials with control groups, is required to validate and expand upon these findings. In addition, children with ASD may present not only with amblyopia but also with strabismus and difficulties in eye movements, visual attention span, and visual perceptual skills. Multimodal approaches combining different therapeutic modalities have been reported to be effective for these problems [25, 26]. Future studies should therefore evaluate not only visual acuity but also these broader aspects of visual function.

## 4. Conclusion

In this case, dichoptic treatment using Occlu‐pad resulted in marked improvement in visual acuity and high level of treatment compliance in a child with anisometropic amblyopia and ASD, for whom conventional occlusion therapy was difficult to implement. The Occlu‐pad system requires the use of polarized glasses to view a tablet screen, which serves as a motivating factor by encouraging voluntary and consistent participation in therapy because children must wear the glasses to watch videos or play games. While sensory hypersensitivity and strong preferences commonly observed in children with ASD can interfere with conventional monocular occlusion (i.e., eye patching), binocular treatment using Occlu‐pad may help mitigate such challenges. Therefore, it is a promising alternative in cases where traditional patching is poorly tolerated.

## Funding

No funding was received for this manuscript.

## Consent

Written informed consent was not required, as the patient is sufficiently anonymized in accordance with the ICMJE recommendations and no identifiable information is included.

## Conflicts of Interest

The authors declare no conflicts of interest.

## Data Availability

All data supporting the findings of this study are included in the article.
